# Mid infrared polarization engineering via sub-wavelength biaxial hyperbolic van der Waals crystals

**DOI:** 10.1038/s41598-021-86056-x

**Published:** 2021-03-23

**Authors:** Saurabh Dixit, Nihar Ranjan Sahoo, Abhishek Mall, Anshuman Kumar

**Affiliations:** 1 Laboratory of Optics of Quantum Materials, Department of Physics, IIT Bombay, Mumbai, Maharashtra 400076 India; 2 Max Planck Institute for the Structure and Dynamics of Matter, Luruper Chaussee 149, 22761 Hamburg, Germany

**Keywords:** Optical materials and structures, Mid-infrared photonics

## Abstract

Mid-infrared (IR) spectral region is of immense importance for astronomy, medical diagnosis, security and imaging due to the existence of the vibrational modes of many important molecules in this spectral range. Therefore, there is a particular interest in miniaturization and integration of IR optical components. To this end, 2D van der Waals (vdW) crystals have shown great potential owing to their ease of integration with other optoelectronic platforms and room temperature operation. Recently, 2D vdW crystals of $\alpha$-$\hbox {MoO}_{3}$ and $\alpha$-$\hbox {V}_2 \hbox {O}_5$ have been shown to possess the unique phenomenon of natural in-plane biaxial hyperbolicity in the mid-infrared frequency regime at room temperature. Here, we report a unique application of this in-plane hyperbolicity for designing highly efficient, lithography free and extremely subwavelength mid-IR photonic devices for polarization engineering. In particular, we show the possibility of a significant reduction in the device footprint while maintaining an enormous extinction ratio from $\alpha$-$\hbox {MoO}_{3}$ and $\alpha$-$\hbox {V}_2$ $\hbox {O}_5$ based mid-IR polarizers. Furthermore, we investigate the application of sub-wavelength thin films of these vdW crystals towards engineering the polarization state of incident mid-IR light via precise control of polarization rotation, ellipticity and relative phase. We explain our results using natural in-plane hyperbolic anisotropy of $\alpha$-$\hbox {MoO}_{3}$ and $\alpha$-$\hbox {V}_2$ $\hbox {O}_5$ via both analytical and full-wave electromagnetic simulations. This work provides a lithography free alternative for miniaturized mid-infrared photonic devices using the hyperbolic anisotropy of $\alpha$-$\hbox {MoO}_{3}$ and $\alpha$-$\hbox {V}_2$ $\hbox {O}_5$.

## Introduction

Mid-infrared (mid-IR) region of the electromagnetic spectrum is relevant for several applications^1^ in the area of medical diagnostics^2,3^, thermal imaging^4^, molecular sensing^5^, polarized infrared imaging systems^6,7^ among others. These applications require the generation of polarized IR light and manipulation of the polarization state of IR light. Therefore, there is a particular interest in developing mid-IR components such as sources, detectors, and other opto-electronic components.^8,9^ One of the critical challenges for the development of mid-IR technologies is the miniaturization and integration of conventional optical components with the chip-scale platforms via facile fabrication techniques^10,11^. Optical components for the IR spectral region of 8–$20\,\upmu \hbox {m}$ are relatively less developed than that for IR spectral region of 3–$8\,\upmu \hbox {m}$. For instance, conventional long-wavelength IR (LWIR) polarizers are designed via state of the art lithographic techniques^12^ and exhibit form birefringence with a typical transmission efficiency of 70% and extinction ratio (ER) of 25 dB^13^. Its integration with the chip-scale platform is therefore complex. Besides that, optical components to manipulate angle of the polarization state of incident light, e.g. phase retarders and polarization rotators, are mostly available for IR spectral region up to 10 $\upmu$m^14^. Polarization rotator has been demonstrated using artificial metasurfaces^15^ of graphene nanoribbons and metal wire grids in the IR spectral region up to 10 $\upmu$m which involves the complex state of the art lithography techniques^16–20^. Hence the challenge of manipulating the polarization state of IR light in the spectral region of 10–$20\,\upmu \hbox {m}$ still persists. To this end, the newly discovered van der Waals (vdW) materials can enable a new class of small footprint mid-IR photonic components and their easy integration with conventional platforms through van der Waals integration^11,21–24^.

Hyperbolic materials (HMs) are a class of anisotropic materials that have opposite signs of principal components of the real part of dielectric permittivity tensor^25^. Hence, HMs behave like metal in the one crystal direction and dielectric in the other. This property has enabled several exciting applications such as hyperlens, negative refraction, thermal emission engineering, and other exotic optical devices^26^. While most traditional realizations of HMs have been based on a metamaterial approach where careful engineering of the constituent unit cell enables the characteristic hyperbolic permittivity tensor, such approaches are complicated due to the required nanofabrication and lithography to make the unit cell significantly subwavelength for the effective medium approximation to work^26^.

In recent years, there has been the emergence of a new class of “naturally” hyperbolic materials (NHMs) where the structural anisotropy of the crystal unit cell itself gives rise to a hyperbolic permittivity without need for lithography techniques^27^. Examples of such 2D vdW materials include black phosphorus^18,28^, h-BN^29,30^, $\hbox {WTe}_2$^31,32^, $\hbox {MoTe}_2$^33^, $\alpha$-$\hbox {V}_2$ $\hbox {O}_5$^34^ and $\alpha$-$\hbox {MoO}_3$^35–37^. These systems can be broadly classified as plasmonic or phononic depending the type of quasiparticle that couples with light to produce the anisotropic optical response. Compared to optical phonon based NHMs, several plasmonic based systems (for example, $\hbox {WTe}_2$ and $\hbox {MoTe}_2$, black phosphorus) suffer from large optical losses due to intraband and interband contributions. Among phonon based systems, hBN is the most popular, however it is uniaxial in its optical response, hence not suitable for the type of in-plane anisotropy that we desire for our application. On the other hand, $\alpha$-$\hbox {MoO}_3$ and $\alpha$-$\hbox {V}_2$ $\hbox {O}_5$ are phonon based biaxial hyperbolic materials in the long-wavelength mid-IR and hence are more suitable for designing highly efficient optical components. To the best of our knowledge, the optical properties of these biaxial NHMs have not been explored for their application in polarizer and polarization rotator in the mid-IR spectral region. A summary of recently investigated vdW crystals and their application for IR polarizer and polarizer rotator has been provided in Table 1.

**Table 1:** Overview of various 2D vdW crystals and their applications in the infrared photonic devices, namely polarizer and polarization rotator.

vdW materials	Hyperbolic spectral range	Complexity involved	Polarizer	Polarization rotator
Graphene nanoribbon^18^	100–450 $\hbox {cm}^{-1}$	Requires nano-patterning	Not reported	$-6$°–18°^18^
Black Phosphorus^18^	1200–$2600\,\hbox {cm}^{-1}$	Unstable in air^38^	ER $\ge 9$ dB^39^	0°–10°^18^
h-BN^29^	780–$830\;\hbox {cm}^{-1}$; 1370–$1610\;\hbox {cm}^{-1}$	Requires tilted optical axis or nanopatterning	ER $\ge 14$ dB^40^; T.E. $\ge 90\%$; B/W: $1.15\,\upmu \hbox {m}$	Not reported
$\hbox {WTe}_2$^31,32^	427–623 $\hbox {cm}^{-1}$	Hyperbolic below 200 K temperature^31^	Not reported	Not reported
$\hbox {MoTe}_2$^33^	Tunable in visible and near IR	Requires monolayer	Not reported	Not reported
$\alpha$-$\hbox {MoO}_3^{\mathrm{this\,work}}$	545–850 $\hbox {cm}^{-1}$; 820–972 $\hbox {cm}^{-1}$; 958–1006 $\hbox {cm}^{-1}$	None	ER $\ge 30$ dB; T.E. $\ge$ 70%; B/W: $3.24\,\upmu \hbox {m}$	0°–90°; $62\%\ge \hbox {T.E.}\ge 30\%$; B/W: $0.76\,\upmu \hbox {m}$
$\alpha$-$\hbox {V}_2$ $\hbox {O}_5^{\mathrm{this\,work}}$	506–842 $\hbox {cm}^{-1}$; 765–952 $\hbox {cm}^{-1}$; 976–1037 $\hbox {cm}^{-1}$	None	ER $\ge 30$ dB; T.E. $\ge$ 55%; B/W: $2.84\,\upmu \hbox {m}$	0°–90°; $42\%\ge \hbox {T.E.}\ge 20\%$; B/W: $1.50\,\upmu \hbox {m}$

*ER* extinction ratio, *T.E.* transmission efficiency, *B/W* bandwidth.

Molybdenum trioxide ($\alpha$-$\hbox {MoO}_{3}$) and vanadium oxide ($\alpha$-V${_2}$ $\hbox {O}_5$) are the vdW crystals which have orthorhombic unit cell and exhibit biaxial hyperbolic anisotropy in the mid-IR spectral region of light (see Table 1)^34,37,41,42^. Orthorhombic unit cell structure of $\alpha$-$\hbox {MoO}_{3}$ is composed of distorted $\hbox {MoO}{_6}$ octahedra and has three different lattice constants (*a*, *b*,  and *c*)^41^. Importantly, the in-plane lattice constants of $\alpha$-$\hbox {MoO}_{3}$ have a difference of 7%, resulting in a strong in-plane anisotropy^41^. Similarly, the orthorhombic unit cell of $\alpha$-$\hbox {V}{_2}$ $\hbox {O}_5$ is composed of three in-equivalent oxygen atoms with respect to vanadium atoms with asymmetric V-O bonds in all three crystal directions, which results in biaxial anisotropy. Moreover, fabrication of $\alpha$- $\hbox {MoO}_3$^43,44^ and $\alpha$- $\hbox {V}_2$ $\hbox {O}_5$^45,46^ thin films can be carried out using low-cost techniques like physical vapor deposition and chemical vapor transport techniques yielding thickness ranging from few hundreds of nanometers to few microns. This approach can further be complemented with mechanical exfoliation technique to obtain a variety of thicknesses of these vdW single crystals. Intrinsic in-plane hyperbolic anisotropy of $\alpha$-$\hbox {MoO}_3$ and $\alpha$-$\hbox {V}_2$ $\hbox {O}_5$ vdW crystals can enable highly efficient and small footprint LWIR optical components which we will discuss in this work.

**Figure 1:**
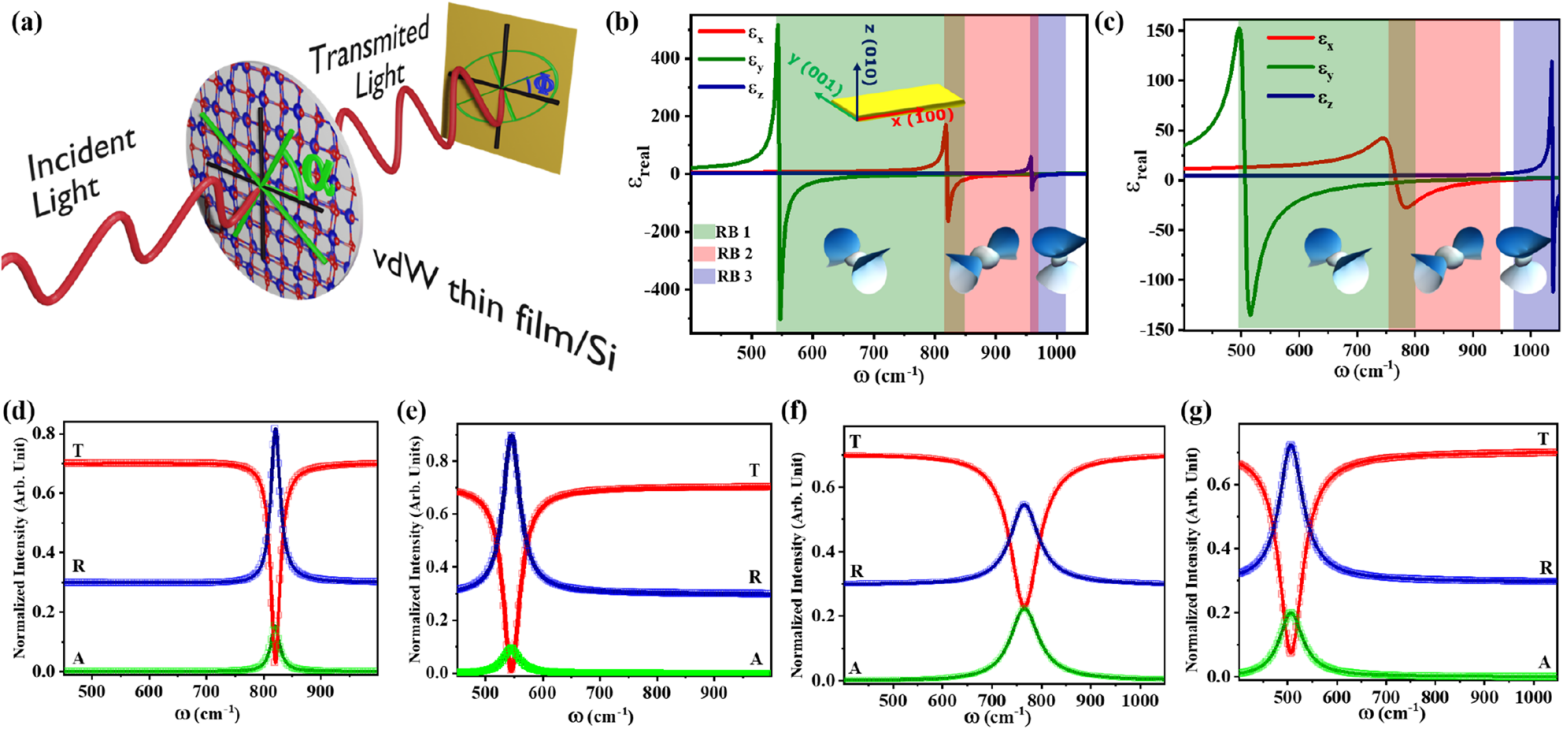
(**a**) Schematic illustration of geometry for vdW thin films (i.e. $\alpha$-$\hbox {MoO}_3$ or $\alpha$-$\hbox {V}_2$ $\hbox {O}_5$) on silicon substrate based polarizer and polarization rotator. (**b**, **c**) show real part of dielectric permittivities of $\alpha$-$\hbox {MoO}_3$ and $\alpha$-$\hbox {V}_2$ $\hbox {O}_5$, respectively, along *x*, *y* and *z* crystallographic direction which reveal the natural biaxial hyperbolicity of $\alpha$-$\hbox {MoO}_{{3}}$ and of $\alpha$-$\hbox {V}_2$ $\hbox {O}_5$ vdW crystals. Spectral region colored in green, red and blue represents Reststrahlen Bands (RBs) 1, 2 and 3 respectively. (Insets show isofrequency surfaces^37^ in the respective RB spectral regions.) (**d**, **e**) represent the optical response of 100 nm thin film of $\alpha$-$\hbox {MoO}_{{3}}$ for $x-$ and *y*-polarized light respectively. (**f**, **g**) represent the optical responses of 100 nm thin film of $\alpha$-$\hbox {V}_2$ $\hbox {O}_5$ for *x*- and *y*-polarized light respectively. Scatter plots and line plots in (**d**–**g**) represent optical responses obtained from numerical simulations and TMM respectively. A, R and T in (**d**–**g**) correspond to absorbance, reflectance and transmittance respectively.

In this report, we propose a lithography free alternative for designing highly efficient mid-IR polarizer and polarization rotator using biaxial NHMs. We have considered a thin film of vdW material, exhibiting natural biaxial hyperbolicity (i.e. $\alpha$-$\hbox {MoO}_3$ and $\alpha$-$\hbox {V}_2$ $\hbox {O}_5$ crystals) in the mid-IR spectral region, on a silicon substrate^47^, and we examine the optical response of these vdW crystals as a function of thickness and frequency. In this work, the excitation or incident IR light intensity is assumed to be low enough so that there is a negligible change in the film temperature. We use the transfer matrix method (TMM)^48,49^ (see Fig. S1 in sec. S1 of supplementary information) and the finite element method based numerical simulation via comsol multiphysics^50^ to evaluate the optical responses of these vdW thin films. We carry out an optimization of standard performance metrics (such as ER, transmission efficiency and range of polarization rotation) for two mid-IR optical devices, namely, polarizer and polarization rotator (schematically shown in Fig. 1a). We find that $\alpha$-$\hbox {MoO}_3$ and $\alpha$-$\hbox {V}_2$ $\hbox {O}_5$ thin films based polarizer exhibit remarkable ER over 30 dB with transmission efficiencies 70% and 55% respectively – characteristics which are attributed to the in-plane hyperbolic anisotropy of these vdW crystals. Moreover, we observe that one can manipulate the polarization state of incident linearly polarized light from 0° to 90° by rotating the principal axes of $\alpha$-$\hbox {MoO}_3$ and $\alpha$-$\hbox {V}_2$ $\hbox {O}_5$ thin films. These studies provide a lithography free^12,51^ alternative for designing the miniaturized and chip-scale mid-IR optical devices using NHMs.

**Figure 2:**
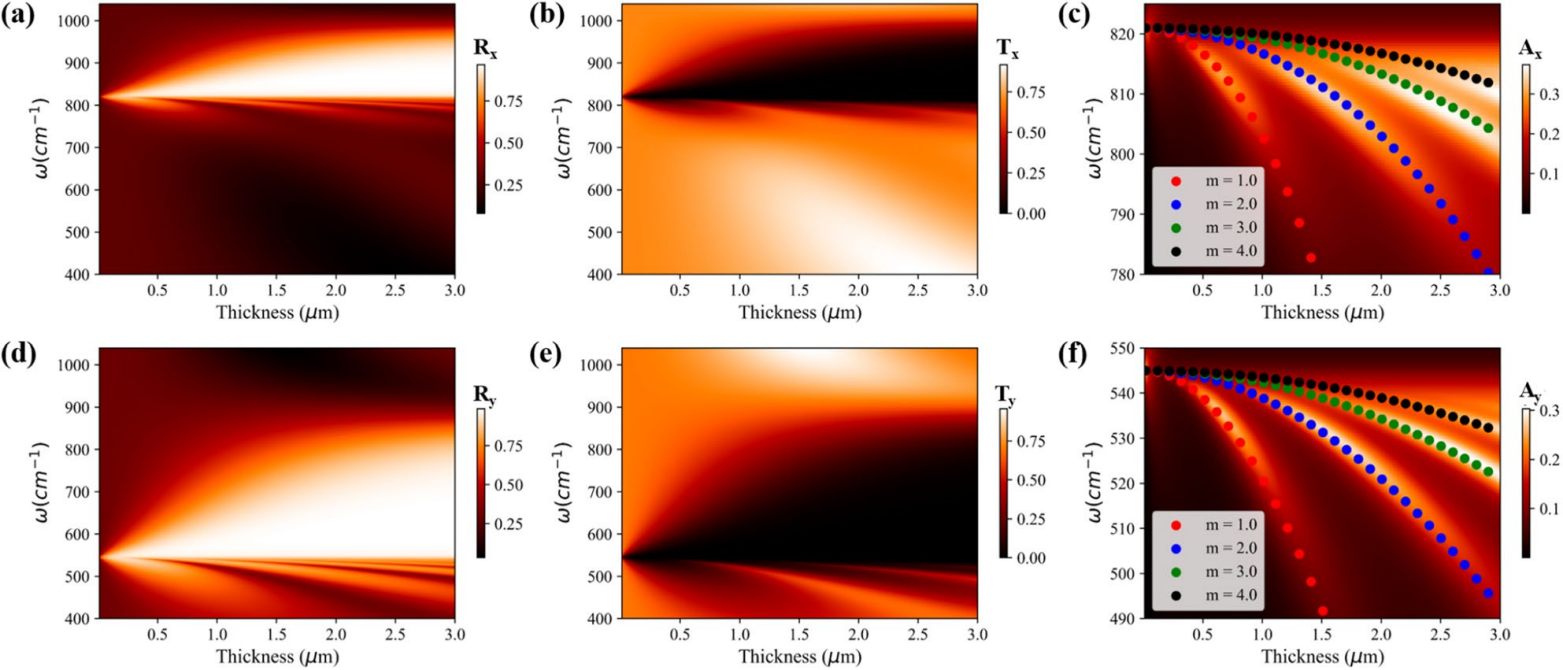
(**a**–**c**) correspond to the reflectance, transmittance, and absorbance, respectively, of $\alpha$-$\hbox {MoO}_{{3}}$ for *x*-polarized incident light as a function of thickness and frequency. (**d**–**f**) represent the reflectance, transmittance, and absorbance, respectively, of $\alpha$-$\hbox {MoO}_{{3}}$ for *y*-polarized incident light as a function of thickness and frequency. The scatter plots in absorption color plots correspond to Fabry Perot modes of $\alpha$-$\hbox {MoO}_{{3}}$ film.

## Results and discussion

Optical responses of $\alpha$-$\hbox {MoO}_3$ and $\alpha$-$\hbox {V}_2$ $\hbox {O}_5$ in the mid-IR spectral region are governed by the optical phonons of the materials and its dielectric permittivity tensor follows the Lorentz model given by^37^:

1 $$\begin{aligned} \varepsilon _{j} = \varepsilon _j^{\infty } \left( 1 + \frac{(\omega _j^{LO})^2 - (\omega _j^{TO})^2}{(\omega _j^{TO})^2 - \omega ^2 - i\omega \Gamma _j}\right) \end{aligned}$$

where *j* represents principal axis direction (i.e. *x*, *y* and *z*) of the $\alpha$-$\hbox {MoO}_{{3}}$ and $\alpha$-$\hbox {V}_2$ $\hbox {O}_5$ crystals. The directions *x*, *y* and *z* correspond to [100], [001] and [010] crystallographic directions respectively. Here $\varepsilon _{j}$ is the principal component of dielectric tensor, whereas $\varepsilon _j^{\infty }$, $\omega _j^{LO}$ and $\omega _j^{TO}$ represent high-frequency dielectric constant, frequency of longitudinal optical (LO) and transverse optical (TO) phonons, respectively in the *j* direction. Lastly, $\Gamma _{j}$ represents the line-width of oscillations in the respective directions. The values of these parameters have been taken from the literature^34,37^ and are tabulated in Sec. S2 of supplementary information. The real part of the principal components of the dielectric tensors of $\alpha$-$\hbox {MoO}_3$ and $\alpha$-$\hbox {V}_2$ $\hbox {O}_5$ are plotted as a function of frequency in Fig. 1b,c respectively which validates the in-plane hyperbolic anisotropy of $\alpha$-$\hbox {MoO}_3$ and $\alpha$-$\hbox {V}_2$ $\hbox {O}_5$ vdW crystals. Dielectric function of $\alpha$-$\hbox {MoO}_3$ in Fig. 1b exhibits three RBs where real part of dielectric permittivity is negative from 545 to 1010 $\hbox {cm}^{-1}$ in atleast one crystallographic direction. Reststrahalen Bands 1–3 lie in the range of 545–850 $\hbox {cm}^{-1}$, 820–973 $\hbox {cm}^{-1}$ and 958–1010 $\hbox {cm}^{-1}$, where dielectric permittivity is negative along *y*, *x* and *z* crystal directions respectively. Similarly, dielectric function of $\alpha$-$\hbox {V}_2$ $\hbox {O}_5$ in Fig. 1c also exhibits three RBs which lies from 506 to 842 $\hbox {cm}^{-1}$, 765 $\hbox {cm}^{-1}$ to 952 and 976 to 1037 $\hbox {cm}^{-1}$, where real part of dielectric permittivity is negative along *y*, *x* and *z* crystal directions respectively. This property fulfills the fundamental criterion of desirable HMs for mid-IR optical components, as explained later in this paper.

### Optical responses of $$\alpha$$-$$\hbox {MoO}_{{3}}$$ and $$\alpha$$-$$\hbox {V}_2$$$$\hbox {O}_5$$ thin films

Using the above dielectric permittivity tensors, we develop our analytical model by transfer matrix method (TMM) for assessing the optical responses of $\alpha$-$\hbox {MoO}_{{3}}$ and $\alpha$-$\hbox {V}_2$ $\hbox {O}_5$. Optical response for *x*-polarized and *y*-polarized incident light on a 100 nm thin film of $\alpha$-$\hbox {MoO}_{{3}}$ are shown in Fig. 1d,e respectively. A sharp dip is observed in the transmittance spectrum of *x*-polarized light (Fig. 1d) around 820 $\hbox {cm}^{-1}$. This is a manifestation of TO phonons of $\alpha$-$\hbox {MoO}_{{3}}$ along [100] direction due to which dielectric permittivity along that direction becomes negative. Hence, it reflects the *x*-polarized light at this particular frequency, as shown reflectance spectrum of Fig. 1d. A similar phenomenon is observed in the optical response for *y*-polarized light where $\alpha$-$\hbox {MoO}_{{3}}$ exhibits high reflectance at the frequency of TO phonons along [001] direction (i.e. 545 $\hbox {cm}^{-1}$) (as shown in Fig. 1e). A small absorption band is also observed by $\alpha$-$\hbox {MoO}_{{3}}$ thin film, which corresponds to optical losses in the dielectric material. Similarly, the optical response of a 100 nm thin film of $\alpha$-$\hbox {V}_2$ $\hbox {O}_5$ are shown in Fig. 1f,g for *x*-polarized and *y*-polarized incident light respectively. Transmission spectra for *x*-polarized and *y*-polarized light exhibit sharp dips at the frequency of TO phonons of $\alpha$-$\hbox {V}_2$ $\hbox {O}_5$ in *x*- and *y*-crystal directions respectively. A relatively broad absorption band, compared to $\alpha$-$\hbox {MoO}_3$ is observed at TO phonon frequencies because $\alpha$-$\hbox {V}_2$ $\hbox {O}_5$ is a lossier vdW material than $\alpha$-$\hbox {MoO}_3$ which is evident from the values of $\Gamma$ in *x*- and *y*-crystal direction of both vdW materials (see Table S1 in sec. S2 of supplementary information). Analytical results for the optical responses of $\alpha$-$\hbox {MoO}_{{3}}$ and $\alpha$-$\hbox {V}_2$ $\hbox {O}_5$ thin films are further substantiated using finite difference frequency domain (FDFD) based numerical simulation and are found in excellent agreement as shown by the scatter plots in Fig. 1d–g. From these optical responses, it is noticeable that thin film of $\alpha$-$\hbox {MoO}_{{3}}$ and $\alpha$-$\hbox {V}_2$ $\hbox {O}_5$ can reflect light with one state of polarization and pass the light with the second state of polarization near the TO phonon frequency.

**Figure 3:**
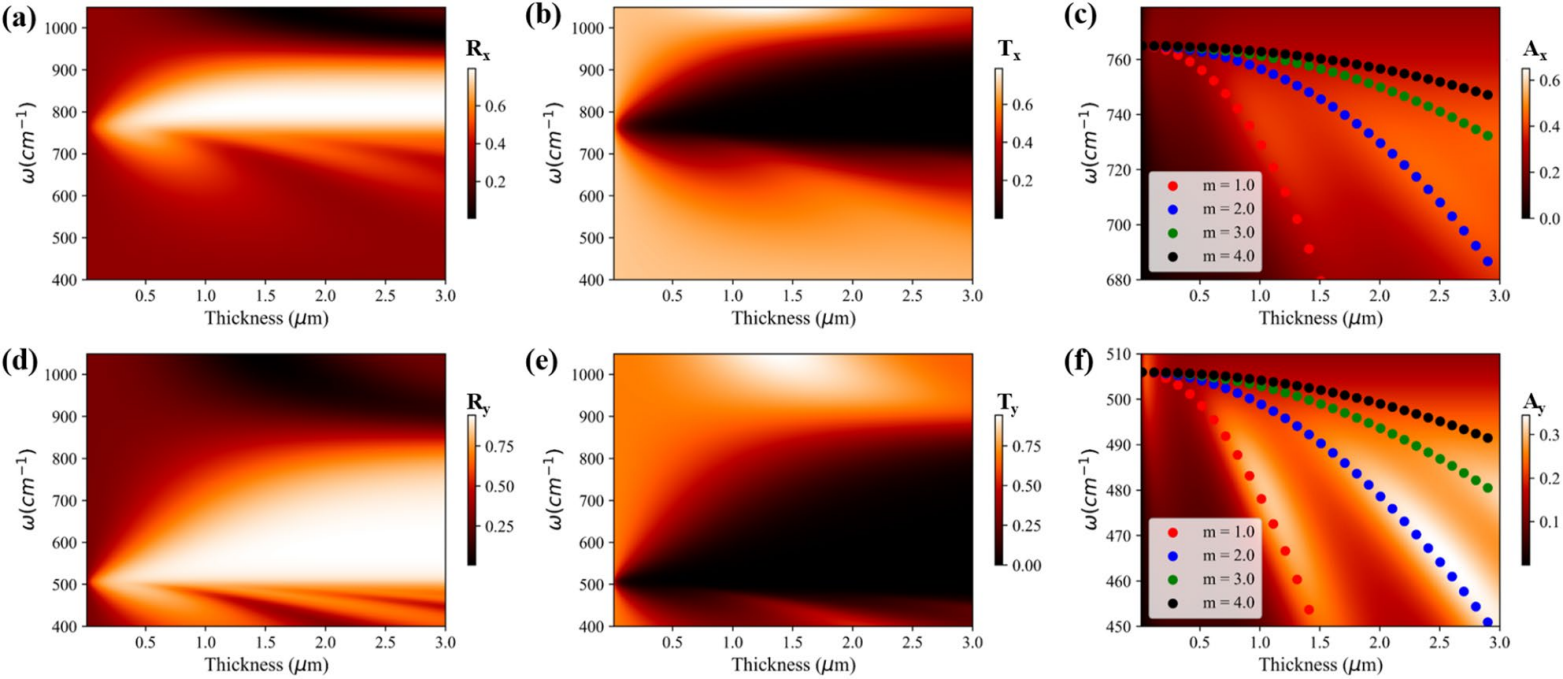
(**a**–**c**) correspond to the reflectance, transmittance, and absorbance, respectively, of $\alpha$-$\hbox {V}_2$ $\hbox {O}_5$ for *x*-polarized incident light as a function of thickness and frequency. (**d**–**f**) represent the reflectance, transmittance, and absorbance, respectively, of $\alpha$-$\hbox {V}_2$ $\hbox {O}_5$ for *y*-polarized incident light as a function of thickness and frequency. The scatter plots in absorption color plots correspond to Fabry Perot modes of $\alpha$-$\hbox {V}_2$ $\hbox {O}_5$ thin film.

Thickness of the vdW crystals is another vital parameter that influences the optical response. Hence, we next investigate the optical responses as a function of the frequency and thickness, as shown in Figs. 2 and 3 for $\alpha$-$\hbox {MoO}_3$ and $\alpha$-$\hbox {V}_2$ $\hbox {O}_5$ respectively. Bandwidth of reflectance for *x*- and *y*-polarized light, within the RB-2 and RB-1 respectively, increases with increasing thickness of $\alpha$-$\hbox {MoO}_3$ (Fig. 2a,d) and $\alpha$-$\hbox {V}_2$ $\hbox {O}_5$ (Fig. 3a,d). This is consistent with increased reflectance from a thicker metallic film. Contrary to reflectance, the bandwidth of transmittance for *x*- and *y*-polarized light in their respective RBs, decreases with the increasing thickness of $\alpha$-$\hbox {MoO}_3$ (Fig. 2b,e) and $\alpha$-$\hbox {V}_2$ $\hbox {O}_5$ (Fig. 3b,e) respectively. Furthermore, few discrete modes are observed in the reflectance color plots which arises below TO phonon frequencies for both *x*- and *y*-polarized light. At the frequencies of dips in the reflectance color plot, strong peaks are observed in the absorbance and transmittance color plot. These features can be clearly seen in the absorbance color plots of $\alpha$-$\hbox {MoO}_3$ (Fig. 2c,f) and $\alpha$-$\hbox {V}_2$ $\hbox {O}_5$ (Fig. 3c,f) for *x*- and *y*-polarized light respectively. Since dielectric permittivity of $\alpha$-$\hbox {MoO}_{{3}}$ and $\alpha$-$\hbox {V}_2$ $\hbox {O}_5$ just below the TO phonon frequency is positive and large, a sub-wavelength thin film of these vdW crystals work as a Fabry–Perot cavity resulting in the observed discrete absorption modes. To investigate these discrete absorption modes, an analytical model is developed (see Sec. S3 of supplementary information) which relates the frequency, thickness, and order of the Fabry Perot mode and given as:

2 $$\begin{aligned} \omega ^2 = \frac{((\omega _j^{LO})^2 + K_j) - \sqrt{((\omega _j^{LO})^2 + K_j)^2 - 4K_j((\omega _j^{TO})^2)}}{2} \end{aligned}$$

Here, $K_j = \frac{1}{\varepsilon _j^\infty } (\frac{m\pi c}{d})^2$, *m* is order of the mode, *c* is speed of light in free space and *d* the thickness of vdW thin film (i.e. $\alpha$-$\hbox {MoO}_{{3}}$ and $\alpha$-$\hbox {V}_2$ $\hbox {O}_5$). The analytical prediction from Eq. (2) (scatter plots), as shown in the Fig. 2c,f for $\alpha$-$\hbox {MoO}_3$ films and Fig. 3c,f for $\alpha$-$\hbox {V}_2$ $\hbox {O}_5$ thin films, for different order of absorption modes of the Fabry-Perot cavity, are in excellent agreement with the discrete modes observed in absorption color plots from our TMM model. Moreover, similar optical responses have been observed for another standard substrate KRS-5 (see Figs. S2 and  S3 in Sec. 4 of supplementary information). Optical responses of $\alpha$-$\hbox {MoO}_{{3}}$ and $\alpha$-$\hbox {V}_2$ $\hbox {O}_5$ thin films suggest that when one component of light is allowed to transmit through these vdW thin films, its orthogonal component of light will be blocked (reflected). Therefore, one can design thin film mid-IR polarizer device via sub-wavelength thin film of these vdW crystals without using any lithography techniques.

### $$\alpha$$-$$\hbox {MoO}_3$$ and $$\alpha$$-$$\hbox {V}_2$$$$\hbox {O}_5$$ thin film based Mid-infrared polarizer

With the above given understanding of the polarization-dependent optical responses of $\alpha$-$\hbox {MoO}_3$ and $\alpha$-$\hbox {V}_2$ $\hbox {O}_5$ thin films, we first explore their potential for the application to mid-IR polarizers. To characterize such a polarizer, we define two well-known figures of merits—transmission efficiency and ER, the latter being given by the formula:

3 $$\begin{aligned} {ER} = {10\log \frac{T_x}{T_y}} \end{aligned}$$

**Figure 4:**
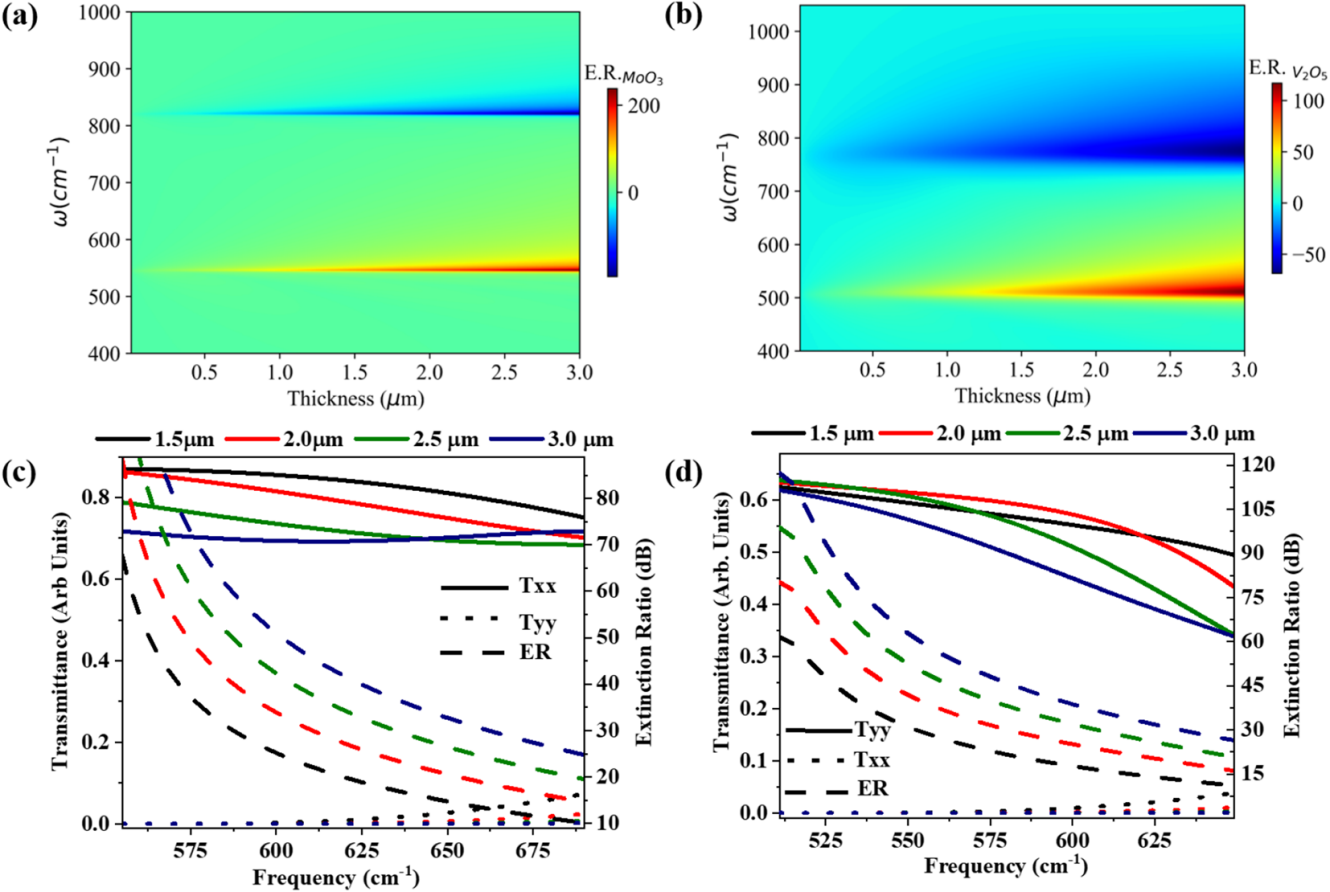
Extinction ratio (in dB) of (**a**) $\alpha$-$\hbox {MoO}_{{3}}$ and (**b**) $\alpha$-$\hbox {V}_2$ $\hbox {O}_5$ based mid-IR polarizer as a function of thickness and frequency. At different thicknesses, figures of merit (i.e. transmission efficiencies and ER) of (**c**) $\alpha$-$\hbox {MoO}_{{3}}$ and (**d**) $\alpha$-$\hbox {V}_2$ $\hbox {O}_5$ based polarizers.

Here, $T{_x}$ and $T{_y}$ are the transmittances of *x*-polarized and *y*-polarized light respectively. ER of $\alpha$-$\hbox {MoO}_3$ and $\alpha$-$\hbox {V}_2$ $\hbox {O}_5$ based polarizers in the transmission mode is shown in Fig. 4a,b respectively as a function of thickness and frequency. For a 1.5–3 $\upmu \hbox {m}$ thin film of $\alpha$-$\hbox {MoO}_3$, we observe an enormous ER of around 200 dB and − 190 dB at the frequency of 546 $\hbox {cm}^{-1}$ and 820 $\hbox {cm}^{-1}$ respectively. Similarly, a thin film of $\alpha$-$\hbox {V}_2$ $\hbox {O}_5$ (i.e. 1.5–3.0 $\upmu$m) exhibits ER around 120 dB and 60 dB at the frequency of 506 $\hbox {cm}^{-1}$ and 765 $\hbox {cm}^{-1}$ respectively. At the TO phonon frequencies of $\alpha$-$\hbox {MoO}_3$ and $\alpha$-$\hbox {V}_2$ $\hbox {O}_5$, we observe enormous ER compared to other frequencies in RBs which is attributed to highly negative dielectric permittivity at TO phonon frequencies. Difference in the ERs of $\alpha$-$\hbox {MoO}_3$ and $\alpha$-$\hbox {V}_2$ $\hbox {O}_5$ thin films at TO phonon frequencies is ascribed to difference in the magnitude of dielectric permittivity of both vdW crystals.

Next, we estimate the operational bandwidth of mid-IR polarizer for various thicknesses of $\alpha$-$\hbox {MoO}_3$ and $\alpha$-$\hbox {V}_2$ $\hbox {O}_5$ in RB-1, shown in the Fig. 4c,d respectively, by considering transmission efficiency and ER thresholds as figures of merits. A mid-IR polarizer based on 3 $\upmu \hbox {m}$ thin film of $\alpha$-$\hbox {MoO}_{{3}}$ exhibits a maximum spectral bandwidth of 166 $\hbox {cm}^{-1}$ (4.28 $\upmu$m) and 117 $\hbox {cm}^{-1}$ (3.24 $\upmu \hbox {m}$) from 545 $\hbox {cm}^{-1}$ at ER thresholds of 20 dB and 30 dB respectively (shown in Fig. 4c) with more than $70\%$ transmission efficiency. Furthermore, a 2–$2.5\,\upmu \hbox {m}$ thin film of $\alpha$-$\hbox {V}_2$ $\hbox {O}_5$ based mid-IR polarizer exhibits a maximum spectral bandwidth of 112 $\hbox {cm}^{-1}$ (3.65 $\upmu \hbox {m}$) and 83 $\hbox {cm}^{-1}$ (2.84 $\upmu$m) from 501 $\hbox {cm}^{-1}$ at the ER thresholds of 20 dB and 30 dB respectively (shown in Fig. 4d) if threshold transmittance efficiency is considered to be $55\%$. The performance metrics of $\alpha$-$\hbox {MoO}_{{3}}$ and $\alpha$-$\hbox {V}_2$ $\hbox {O}_5$ based polarizers are tabulated in Table S2 in Sec. S4 of the supplementary information for other thicknesses. Significant difference between the transmission efficiency of $\alpha$-$\hbox {V}_2$ $\hbox {O}_5$ and $\alpha$-$\hbox {MoO}_{{3}}$ thin film based polarizers is due to large optical losses in the $\alpha$-$\hbox {V}_2$ $\hbox {O}_5$ crystal than $\alpha$-$\hbox {MoO}_3$ crystal. Therefore, $\alpha$-$\hbox {V}_2$ $\hbox {O}_5$ based mid-IR polarizer is slightly less efficient in comparison with that of $\alpha$-$\hbox {MoO}_3$ based mid-IR polarizer. Furthermore, we investigate the figure of merits of these vdW thin film based polarizer in RB-2 spectral region. We conclude that both $\alpha$-$\hbox {MoO}_3$ and $\alpha$-$\hbox {V}_2$ $\hbox {O}_5$ thin film based polarizer are inefficient in the RB-2 spectral region due to their poor transmission efficiency and ER (see Fig. S4 in sec. S4 of supplementary information).

Moreover, we also explore these vdW thin films based mid-IR polarizer in reflection mode and found that they exhibit relatively smaller ER (i.e. $\le$ 10 dB) in the RB-1 and RB-2 (see Fig. S5 of sec. S5 in supplementary information) due to contribution of more than 30% reflectance by orthogonal component of light. We find similar characteristics of polarizer for other standard substrates like KRS-5 (see Fig. S6 in sec. S6 of supplementary information). In summary, Fig. 4 confirms that one can design an excellent mid IR polarizer in transmittance mode using a sub-wavelength thin film of $\alpha$-$\hbox {MoO}_{{3}}$ and $\alpha$-$\hbox {V}_2$ $\hbox {O}_5$ without using any complex lithographic patterning. However, inherent optical losses of $\alpha$-$\hbox {V}_2$ $\hbox {O}_5$ results in lesser transmission efficiency than $\alpha$-$\hbox {MoO}_3$ for polarizer device while sustaining ER of more than 30 dB.

### $$\alpha$$-$$\hbox {MoO}_{{3}}$$ and $$\alpha$$-$$\hbox {V}_2$$$$\hbox {O}_5$$ thin film based mid-IR rotator

Next, we investigate whether the unique in-plane hyperbolicity of these vdW thin films can help achieve polarization engineering in the mid-IR light. We consider a horizontally polarized incident light on these thin films and observe the phase difference, angle of the polarization ellipse axis with respect to the $x-\hbox {axis}$ and ellipticity of transmitted light as a function of frequency and rotation angle of these thin films ($\alpha$) with respect to x-axis. Thickness of the $\alpha$-$\hbox {MoO}_{{3}}$ and $\alpha$-$\hbox {V}_2$ $\hbox {O}_5$ film is taken to be 1.1 $\upmu \hbox {m}$ and 1.0 $\upmu \hbox {m}$ respectively. Angle of the polarization state ($\phi$) and ellipticity ($\sqrt{e}$) are evaluated using the relations^28,52^:

4 $$\begin{aligned} \tan (2\phi )= & {} \frac{-2\cdot M_x\cdot M_y\cdot \cos {\delta }}{{M_x}^2 - {M_y}^2} \end{aligned}$$

5 $$\begin{aligned} \sqrt{e}= & {} \frac{1 + s\sqrt{1 - 4\sin ^2{\delta }\cdot \frac{M_x^2 M_y^2}{(M_x^2 + M_y^2)^2}}}{1 - s\sqrt{1 - 4\sin ^2{\delta }\cdot \frac{M_x^2 M_y^2}{(M_x^2 + M_y^2)^2}}} \end{aligned}$$

**Figure 5:**
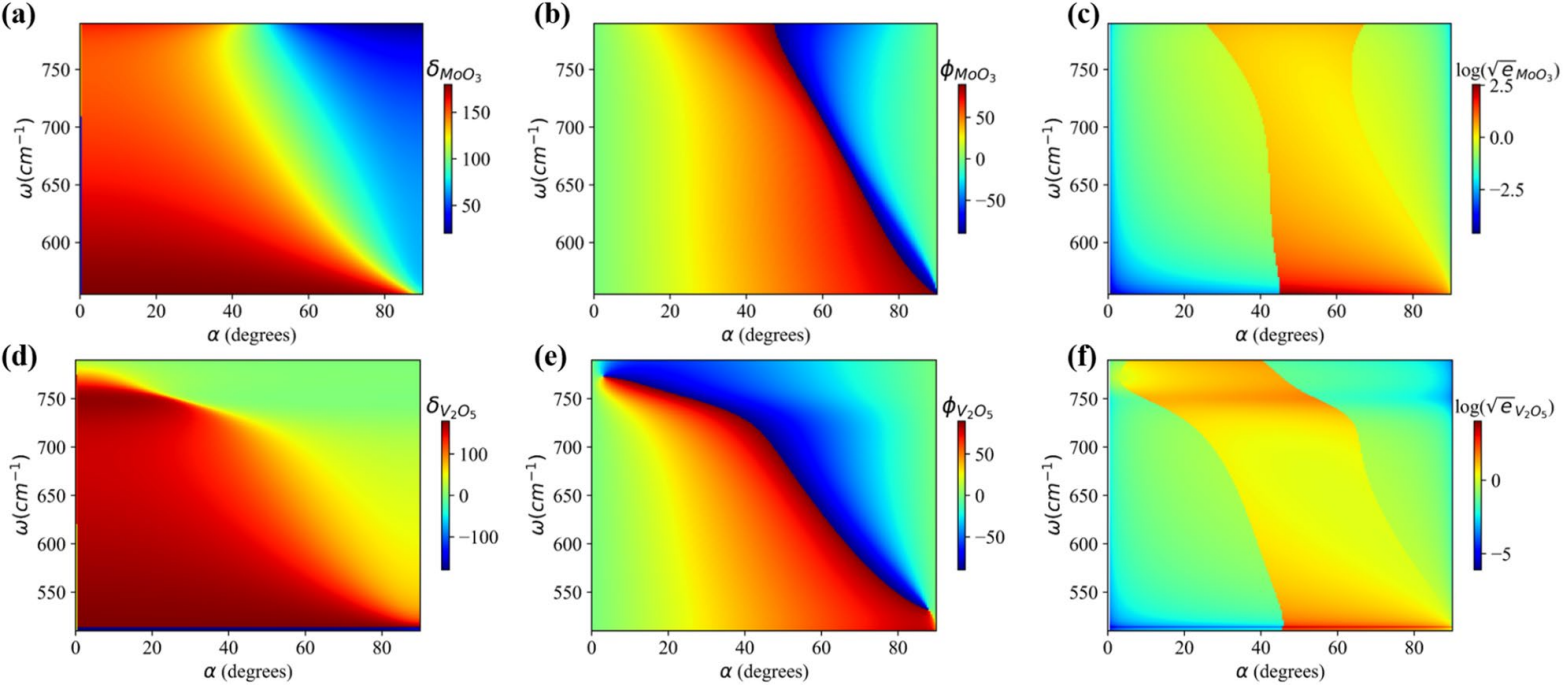
Phase difference ($\delta$) between *x*- and *y*-components, angle of the polarization state ($\phi$) and logarithmic ellipticity ($\log (\sqrt{e})$) of transmitted light through (**a**–**c**) $\alpha$-$\hbox {MoO}_{{3}}$ thin film and (**d**–**f**) $\alpha$-$\hbox {V}_2$ $\hbox {O}_5$ thin film, respectively, as a function of frequency ($\omega$) and rotation angle ($\alpha$).

Here, $M_x$ and $M_y$ are the absolute value of transmission coefficients for *x*- and *y*-components of transmitted light. $s = -1$ when $M_y^2 - M_x^2 < 0$ otherwise $s = 1$ and $\delta = \delta _x - \delta _y$ represents the phase difference between *x* and *y* components of transmitted light. To interpret the amplitude of transmitted light, we have considered the logarithmic ellipticity in the color plots which implies that $\log (\sqrt{e}) = 0$ represents circularly polarized light, whereas elliptically/linearly polarized light for other values. Phase difference between both components of transmitted light, angle of the polarization state and logarithmic ellipticity of transmitted light from these vdW thin films are also substantiated with finite element method one example of which has been shown in the Fig. S7 of sec. S7 in supplementary information. Phase difference as a function of frequency and rotation angle ($\alpha$) of these vdW thin films are shown in Fig. 5a,d respectively. Variation of phase difference is attributed to the in-plane hyperbolic birefringence of these NHMs. Angle of the polarization state of transmitted light as a function of frequency and rotation angle ($\alpha$) (shown in Fig. 5b,e) for these thin films, respectively, reveals their potential in the application of polarization rotator without using any complex lithographic patterning. It suggests that one can engineer the polarization state of incident light in the range of 0°–90° in their respective RB-1 spectral region via rotation of $\alpha$-$\hbox {MoO}_{{3}}$ and $\alpha$-$\hbox {V}_2$ $\hbox {O}_5$ thin films. Logarithmic ellipticity ($\log (\sqrt{e})$) of the transmitted light through $\alpha$-$\hbox {MoO}_{{3}}$ and $\alpha$-$\hbox {V}_2$ $\hbox {O}_5$ thin films, as shown in Fig. 5c,f respectively, in the RB -1 reveals that the transmitted light is elliptically polarized almost throughout the band.

Total transmittance of polarization rotator based on these vdW thin films has been shown in Fig. S8 of the supplementary information as a function of frequency and rotation angle of flake. It can be inferred that $\alpha$-$\hbox {MoO}_{{3}}$ exhibits better transmittance than $\alpha$-$\hbox {V}_2$ $\hbox {O}_5$ based polarization rotator. Polarization rotators based on $\alpha$-$\hbox {MoO}_{{3}}$ and $\alpha$-$\hbox {V}_2$ $\hbox {O}_5$ thin films exhibit more than 30% and 20% of total transmittance, respectively, for the bandwidth of 750–795 $\hbox {cm}^{-1}$ (760 nm) and from 650 to 700 $\hbox {cm}^{-1}$ (1100 nm), where one can rotate the polarization state from 0° to 90°. However, for rotation of the polarization state up to 45°, $\alpha$-$\hbox {MoO}_{{3}}$ and $\alpha$-$\hbox {V}_2$ $\hbox {O}_5$ thin films based polarization rotators exhibit more than 40% and 30% of total transmittance for the bandwidth ranging from 555 to 795 $\hbox {cm}^{-1}$ (5.45 $\upmu \hbox {m}$) and from 500 to 700 $\hbox {cm}^{-1}$ (5.72 $\upmu \hbox {m}$) respectively. Although $\alpha$-$\hbox {V}_2$ $\hbox {O}_5$ provides larger bandwidth for polarization rotation, it exhibits lesser transmission efficiency as compared to $\alpha$-$\hbox {MoO}_3$. We also investigate the properties of $\alpha$-$\hbox {MoO}_{{3}}$ and $\alpha$-$\hbox {V}_2$ $\hbox {O}_5$ thin film in RB-2 in transmission mode which suggests a small range of variation of $\phi$ in RB-2 and major variation appears near TO phonon frequencies only which can be ascribed to large birefringence near TO phonon frequencies (see Fig. S9 of sec. S7 in supplementary information). Furthermore, we also observe that one can vary $\phi$ using these vdW thin films in reflection mode (see Fig. S10 and S11 in sec. S8 of supplementary information respectively). However, the range of variation is around 10° only. In summary, we show that the rotation of biaxial hyperbolic vdW crystals provides a lithography free alternative for highly efficient transmission mode polarization rotator in RB 1 spectral region as shown in the Fig. 5. $\alpha$-$\hbox {V}_2$ $\hbox {O}_5$ provides larger operational bandwidth than $\alpha$-$\hbox {MoO}_3$ but transmission efficiency of $\alpha$-$\hbox {MoO}_3$ based polarization rotator is better than $\alpha$-$\hbox {V}_2$ $\hbox {O}_5$.

## Conclusion

We investigate the optical responses of recently discovered natural hyperbolic material – $\alpha$-$\hbox {MoO}_{{3}}$ and $\alpha$-$\hbox {V}_2$ $\hbox {O}_5$ – which exhibit strong in-plane birefringence. Sub-wavelength thin films of these vdW materials can be harnessed to design miniaturized optical devices in the mid-IR spectral range without using complex lithography techniques and integrated with conventional platforms. Our analysis and optimization reveal the potential of $\alpha$-$\hbox {MoO}_{{3}}$ and $\alpha$-$\hbox {V}_2$ $\hbox {O}_5$ thin films for application of a high-performance mid-IR polarizer with an ER of more than 30 dB for both vdW materials and transmission efficiency of $70\%$ and $55\%$, respectively, in RB-1 spectral region without the need for lithographic patterning. We also observe the potential of $\alpha$-$\hbox {MoO}_{{3}}$ and $\alpha$-$\hbox {V}_2$ $\hbox {O}_5$ thin-films in their respective RB-1 for the rotation of the polarization state of incident light in the transmission mode from 0° to 90° via rotating the principal axes of $\alpha$-$\hbox {MoO}_{{3}}$ and $\alpha$-$\hbox {V}_2$ $\hbox {O}_5$ thin films. Although inherent optical losses in the $\alpha$-$\hbox {V}_2$ $\hbox {O}_5$ crystal make it less efficient than $\alpha$-$\hbox {MoO}_3$, $\alpha$-$\hbox {V}_2$ $\hbox {O}_5$ thin film provides larger operational bandwidth than $\alpha$-$\hbox {MoO}_5$ thin film for the application of mid-IR polarization rotator. Performance metrics of these vdW thin films in transmission mode for polarizer and polarization rotator devices have not been found to be efficient in the RB-2 spectral region compared to that in RB-1. Moreover, polarizer and polarization rotator based on these vdW thin films in reflection mode are less efficient than transmission mode. Our analysis opens up new avenues for lithography free approaches to design the mid-IR optical components using recently explored biaxial hyperbolic vdW crystals.

## Supplementary Information

Supplementary Information 1.

## Data Availability

The datasets generated during and/or analysed during the current study are available from the corresponding author on reasonable request.
