# Supplementary material for: Mid infrared polarization engineering via sub-wavelength biaxial hyperbolic van der Waals crystals
Source: Sci Rep. 2021 Mar 23;11:6612. doi: 10.1038/s41598-021-86056-x (PMC7988130; doi:10.1038/s41598-021-86056-x)
Supplement: Supplementary file 1 — Supplementary Information 1. [file 41598_2021_86056_MOESM1_ESM.pdf]

Mid-infrared polarization engineering via sub-wavelength biaxial hyperbolic van der Waals crystals: Supplementary Information

Saurabh Dixit[1], Nihar Ranjan Sahoo[1], Abhishek Mall[1,2], Anshuman Kumar[1]

*[1]Laboratory of optics of quantum materials, Department of physics
Indian Institute of Technology Bombay, Mumbai 400076, Maharashtra, India*

[2]Max Planck Institute for the Structure and Dynamics of Matter, Luruper Chaussee 149, 22761 Hamburg, Germany

S1: Transfer Matrix Method:

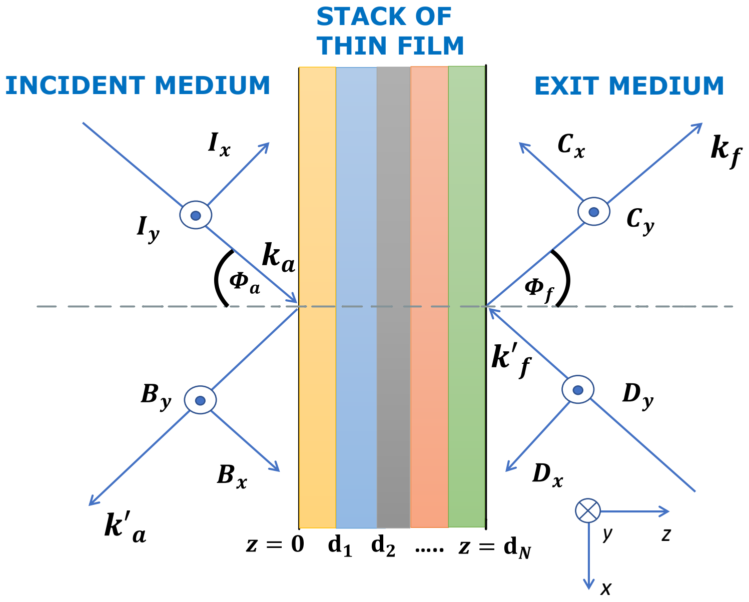

Figure S1: Incident, Reflected, and Transmitted x- and y- polarized component of the plane wave.

Transfer matrix method is a well-established method for calculating optical responses from the stack of thin films on a substrate (Fig.S1).[1,2] As shown in schematic S1, I_x and I_y are the amplitude of incident x- and y- polarized electromagnetic waves. B_x and B_y are the amplitude of reflected x- and y- polarized electromagnetic waves. The stack of N thin films is located at a semi-infinite silicon substrate. C_x and C_y are two components of transmitted electromagnetic waves. In contrast, D_x and D_y will be zero as there is no illumination of light from the backside and no standing waves formation. The four components of light in the incident medium can be related to two components of transmitted light in the exit medium through a general transfer matrix described as:

$$\begin{bmatrix} I_y \\ B_y \\ I_x \\ B_x \end{bmatrix} = \boldsymbol{T} \begin{bmatrix} C_y \\ D_y \\ C_x \\ D_x \end{bmatrix} = \begin{bmatrix} T_{11} & T_{12} & T_{13} & T_{14} \\ T_{21} & T_{22} & T_{23} & T_{24} \\ T_{31} & T_{32} & T_{33} & T_{34} \\ T_{41} & T_{42} & T_{43} & T_{44} \end{bmatrix} \begin{bmatrix} C_y \\ 0 \\ C_x \\ 0 \end{bmatrix} \tag{S1}$$

This 4 X 4 matrix algebra describes the monochromatic plane wave propagation through the entire layer system. The incident and transmitted electromagnetic waves, for i_{th} layer of material with d_i thickness, can be connected with partial transfer matrix $\mathbf{T_{ip}}$ so that the ordered product of all $\mathbf{T_{ip}}$ corresponds to the transfer matrix **T**. Furthermore, an incident matrix ($\mathbf{L_a}$) represents the projection of in-plane component of the incident and reflected electromagnetic waves at the first interface. Likewise, $\mathbf{L_f}$ represents the amplitude of transmitted light from the last interface. Therefore, one can write the transfer matrix **T** as:

$$\boldsymbol{T} = \boldsymbol{L_a^{-1}} \left[\prod_{i=1}^{i=N} \boldsymbol{T_{ip}}\,(d_i) \right]^{-1} \boldsymbol{L_f} \qquad \text{(S2)}$$

The value of $\mathbf{L_a}$, $\mathbf{T_{ip}}$, and $\mathbf{L_f}$ can be obtained from the solution of Maxwell's equations at the respective interfaces given as:

$$\boldsymbol{L_f} = \begin{bmatrix} 0 & 0 & \cos(\emptyset_f) & 0 \\ 1 & 0 & 0 & 0 \\ -n_f cos(\emptyset_f) & 0 & 0 & 0 \\ 0 & 0 & n_f & 0 \end{bmatrix} \quad \&$$

$$\boldsymbol{L_a^{-1}} = \begin{bmatrix} 0 & 1 & \frac{-1}{n_a \cos(\emptyset_a)} & 0 \\ 0 & 1 & \frac{1}{n_a \cos(\emptyset_a)} & 0 \\ \frac{1}{\cos(\emptyset_a)} & 0 & 0 & \frac{1}{n_a} \\ \frac{-1}{n_a \cos(\emptyset_a)} & 0 & 0 & \frac{1}{n_a} \end{bmatrix} \qquad \text{(S3)}$$

where n_a and n_f are the refractive indices of the incident and exit medium (Air and Silicon in our case), $\emptyset_a$ represent the angle of incidence, and $\cos\emptyset_f = \sqrt{\left(1 - \left[\frac{n_a}{n_f}\sin(\emptyset_a)\right]^2\right)}$.

Similarly, the solution of Maxwell equations at the interface of multilayer will provide

$$T_{ip} = \exp(i\frac{\omega}{c} d_i \Delta)\ ;\ \Delta = \begin{bmatrix} k_x \frac{\varepsilon_{31}}{\varepsilon_{33}} & -k_x \frac{\varepsilon_{32}}{\varepsilon_{33}} & 0 & 1 - \frac{k_x^2}{\varepsilon_{33}} \\ 0 & 0 & -1 & 0 \\ -\varepsilon_{21} + \varepsilon_{23}\frac{\varepsilon_{31}}{\varepsilon_{33}} & k_x^2 - \varepsilon_{22} + \varepsilon_{23}\frac{\varepsilon_{32}}{\varepsilon_{33}} & 0 & k_x \frac{\varepsilon_{23}}{\varepsilon_{33}} \\ \varepsilon_{11} - \varepsilon_{13}\frac{\varepsilon_{31}}{\varepsilon_{33}} & \varepsilon_{12} - \varepsilon_{13}\frac{\varepsilon_{32}}{\varepsilon_{33}} & 0 & -k_x \frac{\varepsilon_{13}}{\varepsilon_{33}} \end{bmatrix} \qquad \text{(S4)}$$

where $k_x = n_a \sin(\emptyset_a)$. Using the element of transfer matrix, one can estimate the transmittance (t_x and t_y) and reflectance coefficients (r_x and r_y) from the following set of equations:

$$
\begin{aligned}
r_{yy} &= \left(\frac{B_y}{I_y}\right) = \frac{T_{21}T_{33} - T_{23}T_{31}}{T_{11}T_{33} - T_{13}T_{31}} & r_{xy} &= \left(\frac{B_y}{I_x}\right) = \frac{T_{11}T_{23} - T_{21}T_{13}}{T_{11}T_{33} - T_{13}T_{31}} \\
r_{xx} &= \left(\frac{B_x}{I_x}\right) = \frac{T_{11}T_{43} - T_{41}T_{13}}{T_{11}T_{33} - T_{13}T_{31}} & r_{yx} &= \left(\frac{B_x}{I_y}\right) = \frac{T_{41}T_{33} - T_{43}T_{31}}{T_{11}T_{33} - T_{13}T_{31}} \\
t_{yy} &= \left(\frac{C_y}{I_y}\right) = \frac{T_{33}}{T_{11}T_{33} - T_{13}T_{31}} & t_{xy} &= \left(\frac{C_y}{I_x}\right) = \frac{-T_{13}}{T_{11}T_{33} - T_{13}T_{31}} \\
t_{xx} &= \left(\frac{C_x}{I_x}\right) = \frac{T_{11}}{T_{11}T_{33} - T_{13}T_{31}} & t_{yx} &= \left(\frac{C_y}{I_y}\right) = \frac{-T_{31}}{T_{11}T_{33} - T_{13}T_{31}}
\end{aligned} \tag{S5}
$$

From these coefficients, we evaluate the following:

Reflectance:	$R_i = \vert r_i\vert^2$
Transmittance:	$T_i = \frac{n_f}{n_a}\vert t_i\vert^2$
Absorbance:	$A_i = 1 - R_i - T_i$
Extinction Ratio:	$10\log_{10}\left(\frac{T_x}{T_y}\right)$
Phase difference:	$\angle t_x - \angle t_y$
Total transmittance:	$\frac{n_f}{n_a}(\vert t_{xx}\vert^2 + \vert t_{xy}\vert)$

S2: Parameters for Dielectric function of α-MoO_3 and α-V_2O_5 vdW crystals:

Table S1 Characteristics parameters for the dielectric function of α-MoO_3 and α-V_2O_5 vdW crystals. [3, 4]

	**Crystal directions**	ε_∞	ω_{LO}	ω_{TO}	Γ
**α-MoO_3**	x [100]	4.0	972.0	545.0	4.0
	y [001]	5.2	850.0	820.0	4.0
	z [010]	2.4	1006.0	958.0	2.0
**α-V_2O_5**	x [100]	6.6	952.0	765.0	40.0
	y [001]	6.1	842.0	506.0	19.0
	z [010]	3.9	1037.0	976.0	2.0

S3: Analytical model for Fabry Perot modes in vdW thin films:

The high value of the real part of principle components of dielectric tensor of selected vdW crystals below the corresponding TO phonon frequencies results in the confinement of large wavelength electromagnetic waves in the sub-diffractional thickness of the film, forming the Fabry-Perot cavity. These Fabry-Perot modes are observed as a dip or a peak in the reflectance (transmittance/absorbance) spectrum. To evaluate these modes of absorption, we consider the fundamental condition of maxima in Transmittance/Absorbance in the Fabry Perot cavity[5]:

$$k_0 n d = m\pi \tag{S6}$$

Here, m, d, n, and k_0 (= ω/c) corresponds to the order of mode, the thickness of film, the refractive index in the particular crystal direction, and free space wave-vector, respectively. Refractive index can be evaluated from the square root of the complex dielectric function predicted by Lorentz oscillator model [3] (Eq.1). Taking Γ = 0 and placing it the Eq. S6, we will get:

$$\frac{\omega \times d\sqrt{\varepsilon_\infty\left(\frac{\omega_{LO}^2 - \omega^2}{\omega_{TO}^2 - \omega^2}\right)}}{c} = m\pi \tag{S7}$$

After squaring and rearranging the equation S7, it forms a quadratic equation given as:

$$\omega^4 - \omega^2(\omega_{LO}^2 + K) + K\omega_{TO}^2 = 0$$

where $K = \frac{1}{\varepsilon_\infty}\left(\frac{m\pi c}{d}\right)^2$, the equation has one feasible physical solution represented by the following equation:

$$\omega^2 = \frac{(\omega_{LO}^2 + K) - \sqrt{\left((\omega_{LO}^2 + K)\right)^2 - 4K\omega_{TO}^2}}{2} \tag{S8}$$

S4: Effect of substrate on the optical response of vdW thin films:

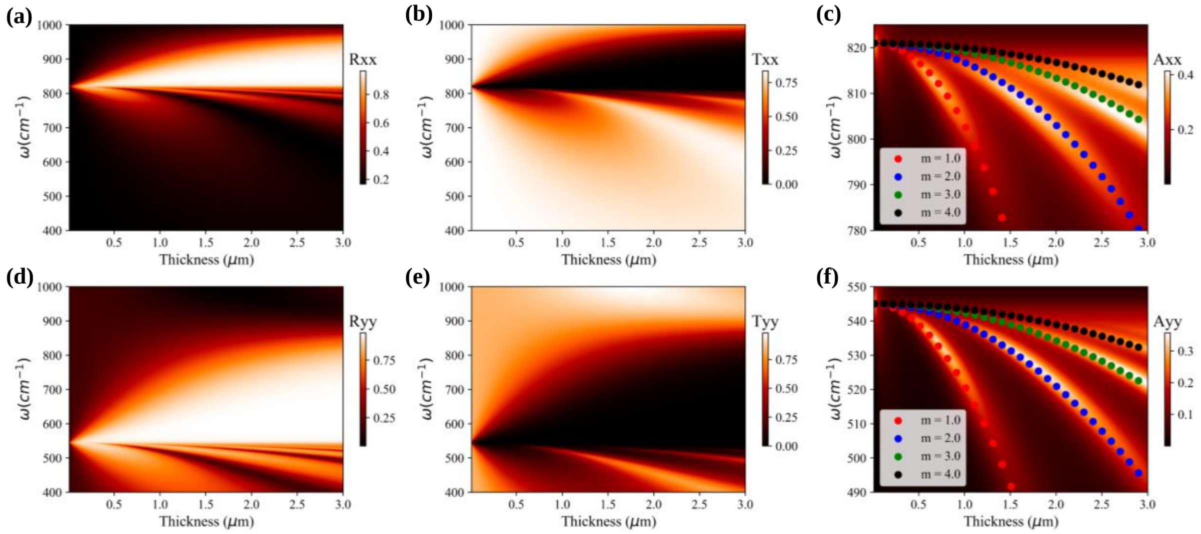

Figure S2 Reflectance, Transmittance and Absorbance of α-MoO3 thin film on KRS-5 substrate[6] for (a)-(c) x-polarized incident light and (d)-(f) y-polarized incident light, respectively. Scatter plots in Fig. (c)&(f) represents Fabry Perot modes of α-MoO_3 thin films.

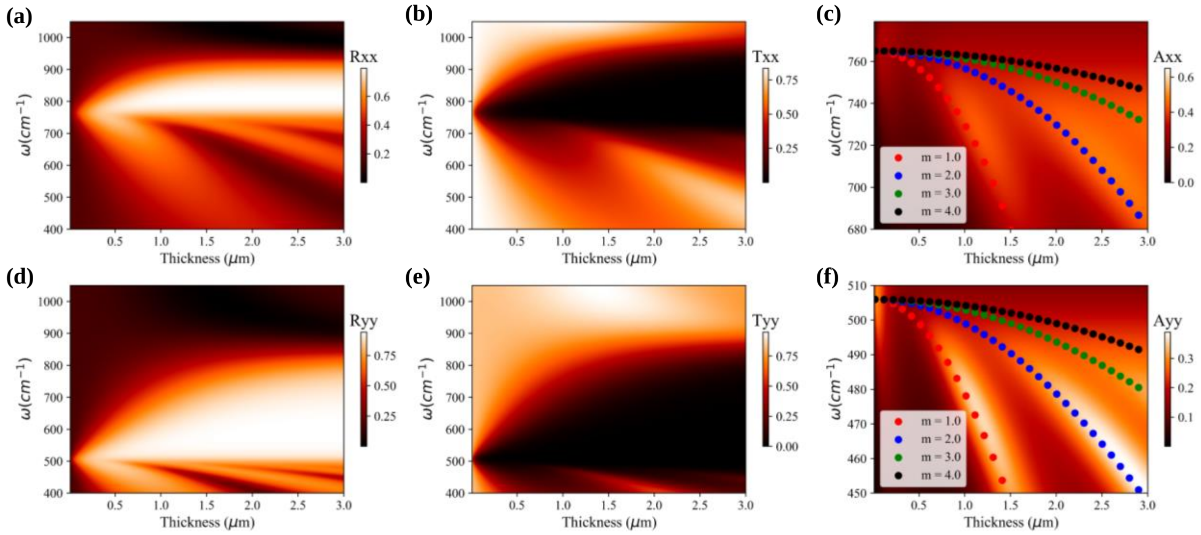

Figure S3: Reflectance, Transmittance and Absorbance of α-V_2O_5 thin film on KRS-5 substrate [6] for (a)-(c) x-polarized incident light and (d)-(f) y-polarized incident light, respectively. Scatter plots in Fig. (c)&(f) represents Fabry Perot modes of α-V_2O_5 thin films.

S4: α-MoO_3 and α-V_2O_5 thin-film based Mid-IR polarizers in transmission mode:

1. Bandwidth of mid-IR polarizers in RB-1:

d (nm)	α-MoO_3				α-V_2O_5			
	ER~ 20 dB		ER ~30 dB		ER~ 20 dB		ER ~30 dB	
	ω (cm⁻¹)	*Bandwidth cm⁻¹(~μm)*	*ω (cm⁻¹)*	*Bandwidth cm⁻¹(~μm)*	*ω (cm⁻¹)*	*Bandwidth cm⁻¹(~μm)*	*ω (cm⁻¹)*	*Bandwidth cm⁻¹(~μm)*
1500	545-619	74 (~2.19)	545-587	43 (~1.31)	501 – 588	87(~2.96)	501 – 552	51 (~1.85)
2000	545- 653	108 (~3.03)	545- 610	65 (~1.96)	**501 – 613**	**112 (~3.65)**	501 – 580	79 (~2.72)
2500	545- 686	141 (~3.77)	545- 635	90 (~2.60)	501 – 584	83 (~2.84)	**501 – 584**	**83 (~2.84)**
**3000**	**545- 711**	**166 (~4.28)**	**545- 662**	**117(~3.24)**	501 – 556	55 (~1.98)	501 – 556	55 (~1.98)

Table S2: Spectral ranges and corresponding bandwidths for the functioning of α-MoO_3 and α-V_2O_5 based mid-IR polarizer in transmission geometry at different thicknesses (d) where transmission efficiency has been considered around 70% α-MoO_3 thin film and 55% for α-V_2O_5 thin film.

2. Mid-IR polarizers figure of merits in RB-2 spectral region:

We explore the possibility of α-MoO_3 and α-V_2O_5 based mid-IR polarizer in their respective RB - 2 spectral region in the transmission mode. We observe that α-MoO_3 and α-V_2O_5 based mid-IR polarizers do not possess remarkable transmission efficiency and extinction ratio in the transmission mode, as shown in Fig. S4(a)&(b). It is ascribed to the differences in the dielectric permittivities along x and y directions. In the RB- 2, α-MoO_3 possess epsilon-near-zero (ENZ) metallic properties in the y-direction and strong metallic properties in the x-direction (see the dielectric function in Fig. 1(b)). Similarly, for α-V_2O_5 based thin film mid-IR polarizer, the transmission efficiency remains less than 55% over the entire RB-2 spectral region (shown in Fig. S4(b)) and hence cannot work as an efficient mid-IR polarizer in RB-2.

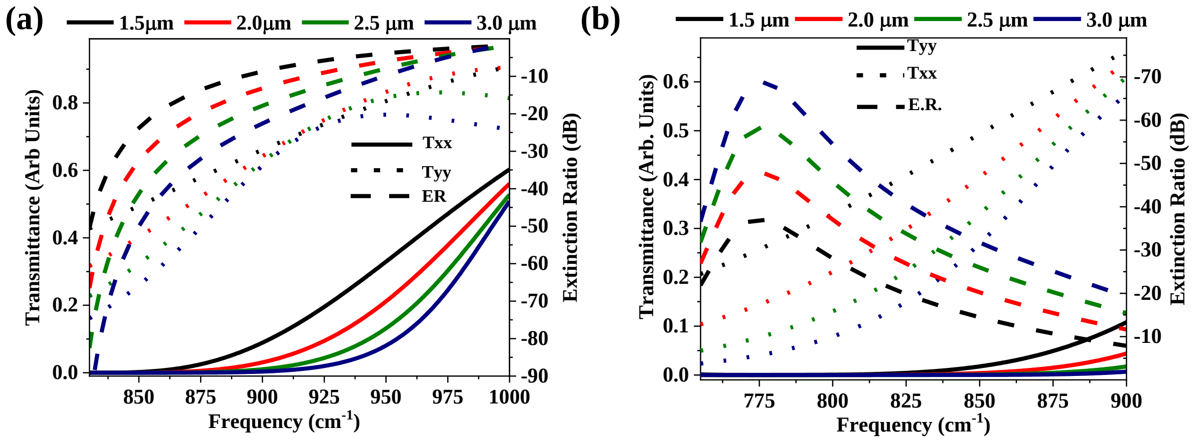

Figure S4: Figure of merits (i.e., transmission efficiency and extinction ratio) for a mid-IR polarizer based on (a) α-MoO3 thin film and (b) α- V_2O_5 thin film at different thicknesses.

S5: α-MoO_3 and α-V_2O_5 based mid-IR polarizer in reflection mode:

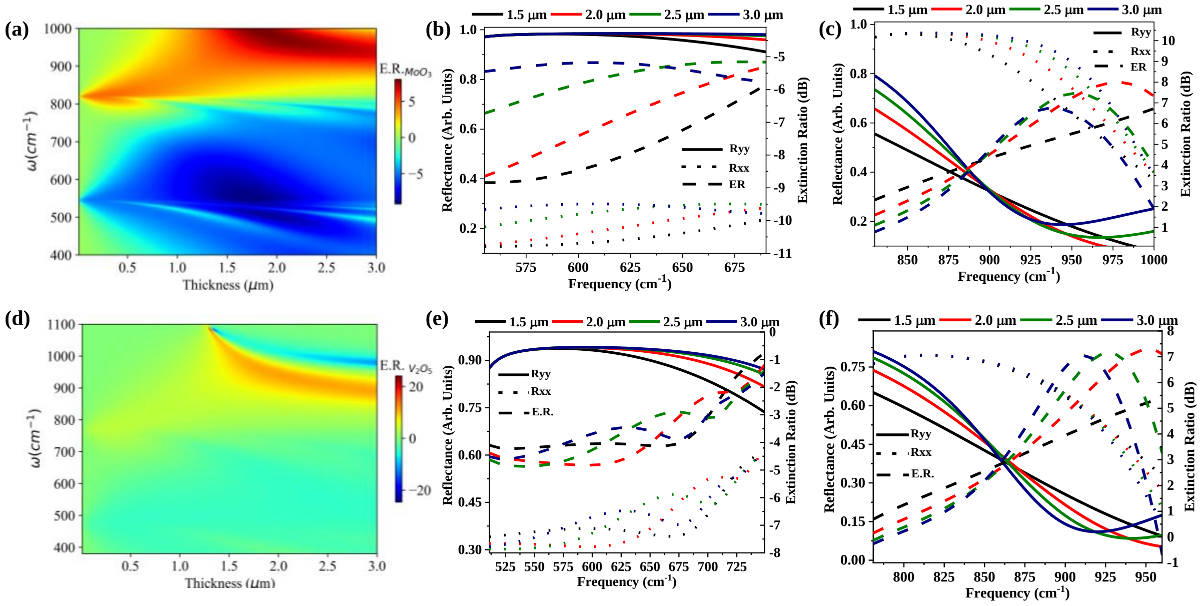

Figure S5: (a) Extinction ratio of α-MoO_3 thin-film based mid-IR polarizer as a function of thickness and frequency. (b) & (c) correspond to the reflection efficiency and ER, respectively, of α-MoO_3 thin-film based mid-IR polarizer at the different thicknesses in RB-1 and RB-2. The α-MoO_3 thin film exhibits relatively less ER (less than 10 dB) in RB-1 and RB-2. Similarly, (d) shows the ER of α-V_2O_5 thin-film based mid-IR polarizer as a function of thickness and frequency. (e) & (f) represents reflection efficiency and ER, respectively, of α-V_2O_5 thin-film based mid-IR polarizer at the different thicknesses in the RB-1 and RB-2.

S6: Effect of substrate on the figure of merits of α-MoO_3 and α-V_2O_5 thin-film based mid-IR polarizer:

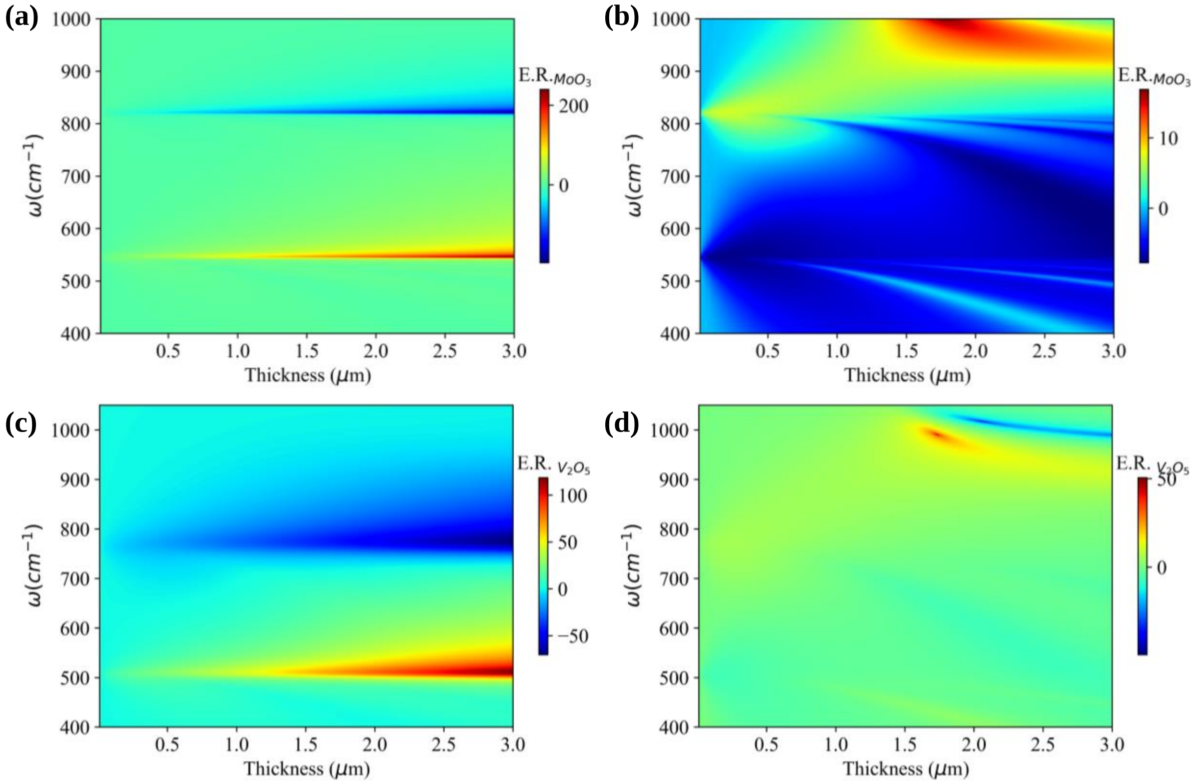

Figure S6: (a)&(b) represent ER of the α-MoO_3 thin film on KRS-5 substrate[6] in the transmission and reflection mode, respectively, as a function of frequency and thickness. Similarly, (c)&(d) represent ER of the α-V_2O_5 thin film on KRS-5 substrate in the transmission and reflection mode, respectively, as a function of frequency and thickness. It is observed that both the vdWs materials exhibit almost similar properties on the KRS-5 substrate and the silicon substrate.

S7: α-MoO_3 and α-V_2O_5 based polarization rotator in transmission mode:

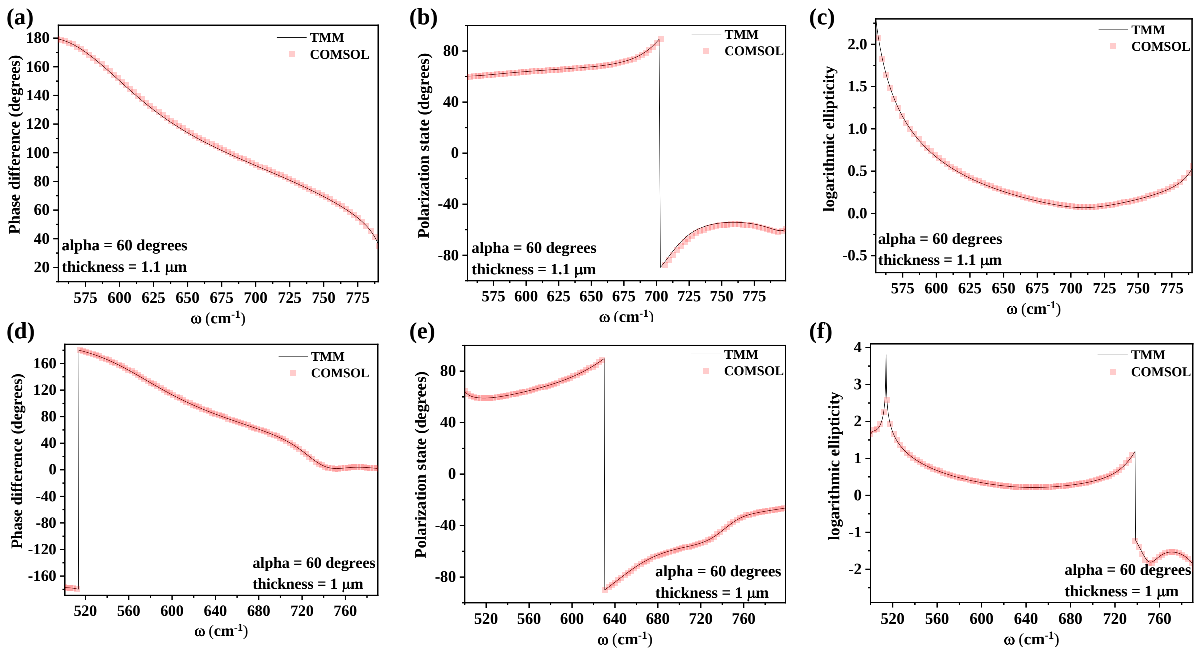

Figure S7 (a)-(c) represent the phase difference between x- and y - components, angle of polarization state (ϕ) and logarithmic ellipticity, respectively, of transmitted light through 1.1 μm thin film of α-MoO_3. Similarly, (d)-(f) represent the phase difference between x- and y - components, angle of polarization state (ϕ) and logarithmic ellipticity, respectively, of transmitted light through 1.0 μm thin film of α-V_2O_5. Here, scatter plots and line plots represent the numerical simulation results (COMSOL) and transfer matrix method results, respectively.

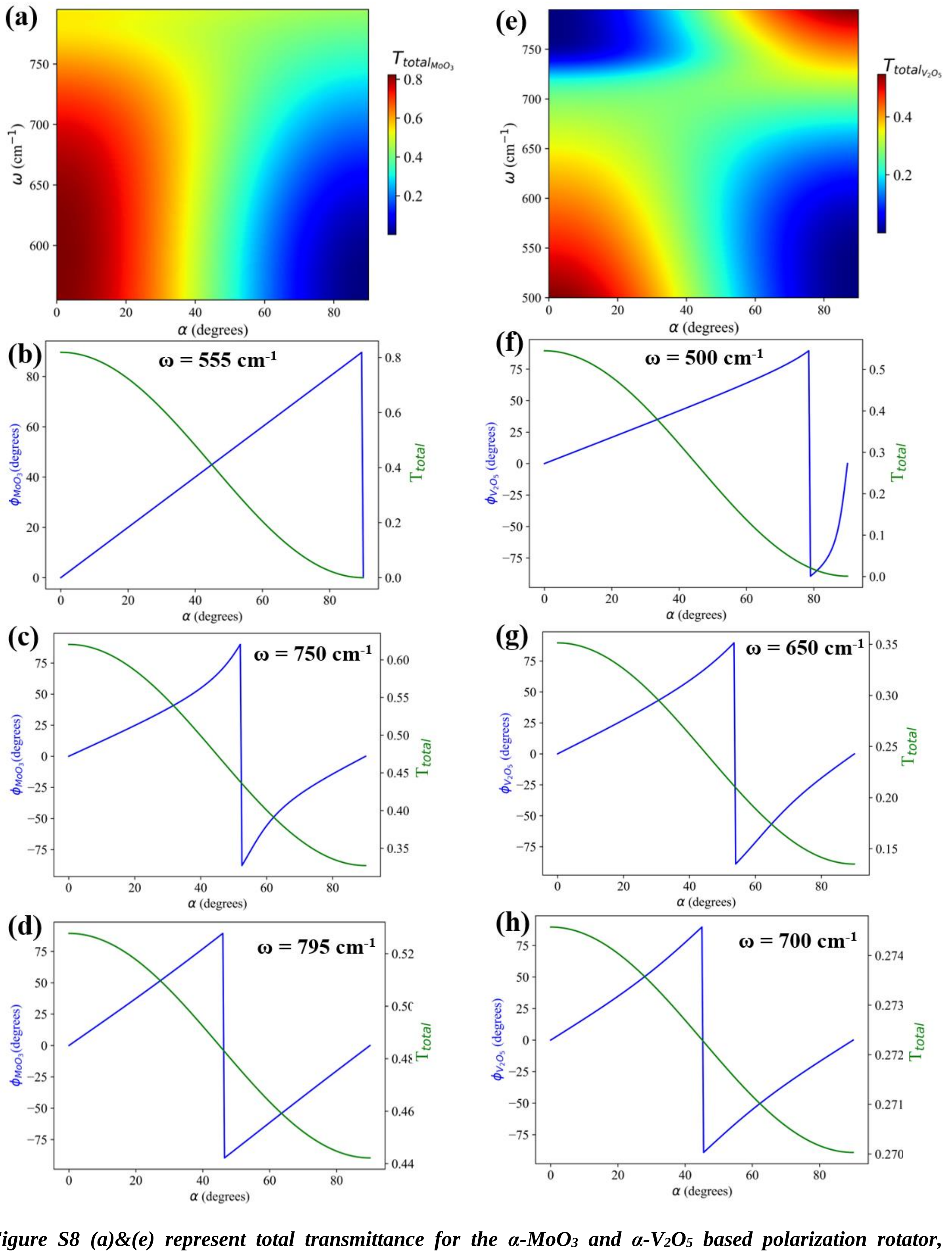

Figure S8 (a)&(e) represent total transmittance for the α-MoO_3 and α-V_2O_5 based polarization rotator, respectively, in transmission mode. (b)-(d) show the total transmittance and angle of the polarization state of α-MoO_3 based polarization rotator as a function of rotation angle at different frequencies. Similarly, (f)-(h) show the total transmittance and angle of the polarization state of α-V_2O_5 based polarization rotator as a function of rotation angle at different frequencies.

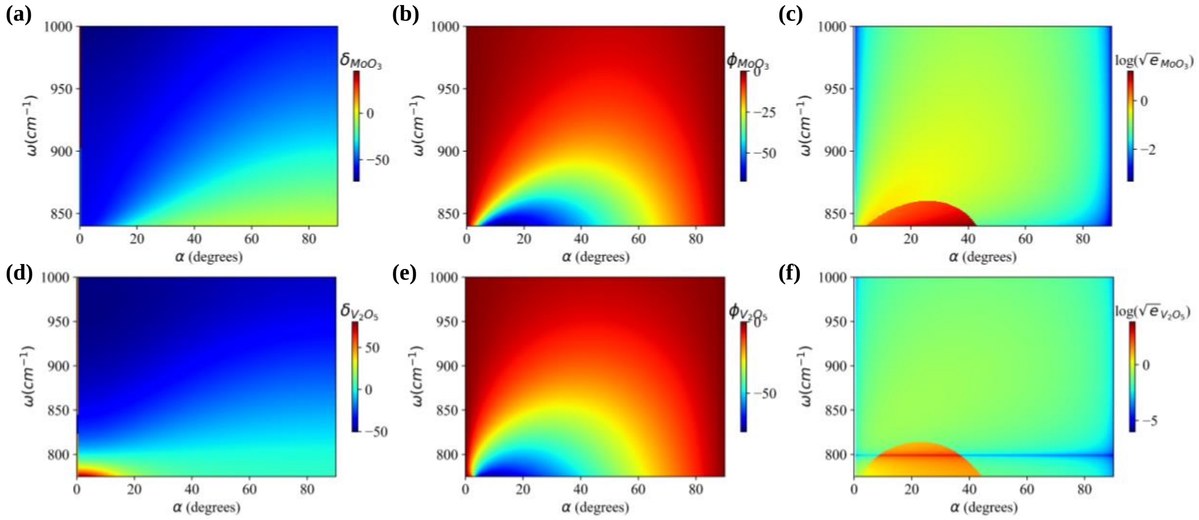

Figure S9: (a)-(c) show phase difference, angle of the polarization state, and ellipticity of transmitted light through 1.1 µm thin-film of α-MoO_3 in the RB-2 spectral region. Similarly, (d)-(f) represent phase difference, angle of polarization state and logarithmic ellipticity of transmitted light through 1.0 µm thin film of α-V_2O_5 in RB-2. For both vdW crystals, angle of the polarization state reveals small variation in the RB-2 spectral region where ellipticity of the transmitted light suggests elliptically polarized light at the output.

S8: α-MoO_3 and α-V_2O_5 based polarization rotator in reflection mode:

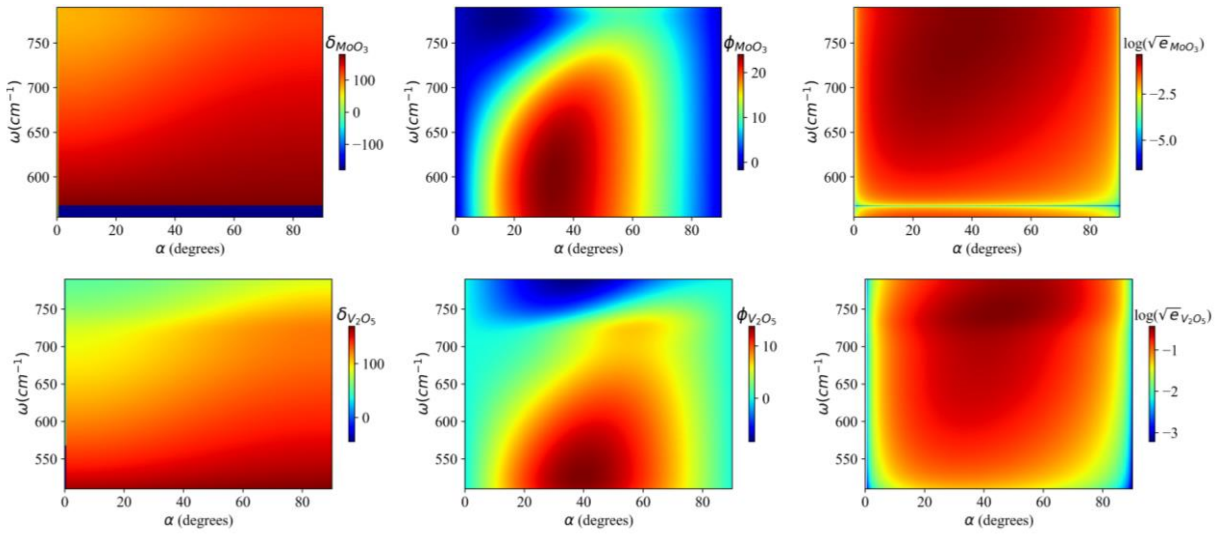

Figure S10 (a)-(c) represent phase difference, angle of polarization state and ellipticity of reflected light, respectively, from 1.1 µm thin-film α-MoO_3 thin film in RB-1 in the reflection mode. Angle of polarization state varies in the range of (0 to 25 degrees) where output is linearly/elliptically polarized light. (d)-(f) represent phase difference, angle of polarization state and ellipticity of reflected light from 1.0 µm thin-film α-V_2O_5 thin film in RB-1. Angle of polarization state varies in the range of 0 degrees to 12 degrees where output is linearly/elliptically polarized light.

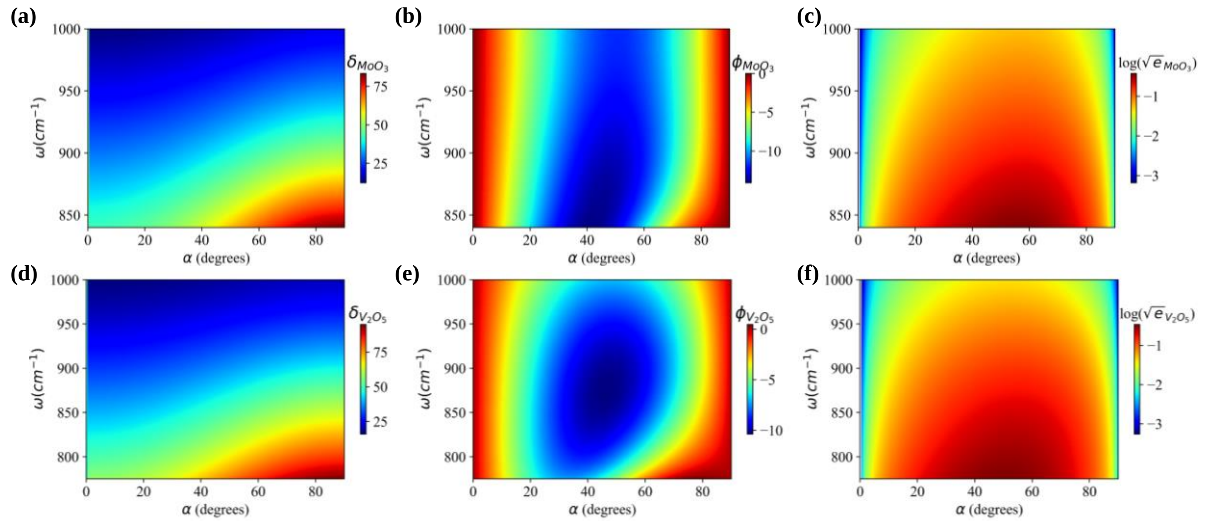

Figure S11 (a)-(c) represents phase difference, angle of polarization state and ellipticity of reflected light from 1.1 µm thin-film α-MoO_3 thin film in RB-2. Angle of polarization state varies in the range of 0 degrees to 15 degrees, where output is linearly polarized light. (d)-(f) represents phase difference, angle of the polarization state and ellipticity of reflected light from 1.0 µm thin-film α-V_2O_5 thin film in RB-2. Angle of polarization state varies in the range of 0 degrees to 10 degrees, where output is linearly/elliptically polarized light

References:

1. Schubert, M. Polarization-dependent optical parameters of arbitrarily anisotropic homogeneous layered systems. *Physical Review B - Condensed Matter and Materials Physics* **53**, 4265–4274 (1996).

2. Passler, N. C. & Paarmann, A. Generalized 4 × 4 matrix formalism for light propagation in anisotropic stratified media: study of surface phonon polaritons in polar dielectric heterostructures. *Journal of the Optical Society of America B* **34**, 2128 (2017).

3. Zheng, Z. *et al.* A mid-infrared biaxial hyperbolic van der Waals crystal. *Science Advances* **5**, 1–9 (2019).

4. Taboada-Gutiérrez, J. *et al.* Broad spectral tuning of ultra-low-loss polaritons in a van der Waals crystal by intercalation. *Nature Materials* 0–5 (2020) doi:10.1038/s41563-020-0665-0.

5. Silfvast, W. T. *Laser Fundamentals*. (Cambridge University Press, 2008).

6. Rodney, W. S. & Malitson, I. H. Refraction and Dispersion of Thallium Bromide Iodide. *Journal of the Optical Society of America* **46**, 956 (1956).
